# Health differences between multiple and single job holders in precarious employment in the Netherlands: A cross-sectional study among Dutch workers

**DOI:** 10.1371/journal.pone.0222217

**Published:** 2019-09-11

**Authors:** Stef Bouwhuis, Goedele A. Geuskens, Cécile R. L. Boot, Allard J. van der Beek, Paulien M. Bongers

**Affiliations:** 1 Amsterdam UMC, Vrije Universiteit Amsterdam, Department of Public and Occupational Health, Amsterdam Public Health Research Institute, Amsterdam, The Netherlands; 2 Netherlands Organisation for Applied Scientific Research TNO, Leiden, The Netherlands; 3 Body@Work, Research Center on Physical Activity, Work and Health, TNO-VU/VUmc, Amsterdam, The Netherlands; London School of Hygiene and Tropical Medicine, UNITED KINGDOM

## Abstract

**Introduction:**

Precarious employment is associated with poor health. Among employees in precarious employment, those with multiple jobs may face additional health risks, e.g. due to combining work schedules and job roles. Our research question is: do differences in health exist between multiple and single job holders in precarious employment?

**Methods:**

Participants in the Netherlands Working Conditions Survey 2012 aged 25–64 years who were not employed through the Act on Social Work Provision and who had a precarious job were included. To select employees in precarious employment (n = 3,609), latent class analysis was performed, based on variables based on indicators described by Van Aerden. Differences in general self-perceived health, burnout complaints, musculoskeletal health, and sickness absence between multiple and single job holders were studied cross-sectionally using logistic regression analyses.

**Results:**

No significant differences were found between multiple and single job holders in precarious employment for self-perceived health (OR = 0.9; 95%CI = 0.7–1.3), burnout complaints (OR = 0.9; 95%CI = 0.7–1.2), and musculoskeletal health (OR = 1.1; 95%CI = 0.8–1.5). In crude analyses, multiple job holders experienced less sickness absence than single job holders (OR = 0.7; 95%CI = 0.5–0.9). In adjusted analyses, this difference was no longer statistically significant (OR = 0.8; 95%CI = 0.6–1.0).

**Conclusions:**

Despite potential health risks related to multiple job holding, we did not find health differences between multiple and single job holders in precarious employment in the Netherlands. More longitudinal research is necessary to provide recommendations for policy makers regarding multiple job holders in precarious employment.

## Introduction

After World War II the ‘standard employment relation’ (SER) became dominant in many western countries. SERs are characterized by stable full-time employment, and protection through collective organization and social rights and benefits [1]. Since the 1970s non-standard work arrangements, such as precarious employment arrangements, have become more widespread [1]. Precarious employment is defined as ‘a state of disempowerment in the employment situation’, resulting in loss of control and insecurity regarding employment conditions and income [2–4]. It refers to a situation in which an employee experiences multiple adverse employment conditions [5]. In most recent research, precarious employment has been associated with a variety of adverse employment conditions, based on the work of Rodgers. He distinguished four dimensions of precariousness: (i) the degree of certainty of continuing work; (ii) degree of control over work, i.e. working conditions; (iii) degree of protection, e.g. against unfair dismissal or unacceptable working practices, for instance regarding occupational health and safety; and (iv) income.

Previous research, mostly cross-sectional, suggests that precarious employment is associated with worse health, e.g. worse general health, occupational injuries, and worse mental health [3,6,7]. Three mechanisms may explain this. First, precarious employment is related to adverse psychosocial experiences, for instance insecurity regarding work and income. Second, precarious employment is related to exposure to low quality working conditions e.g. high physical demands. Third, precarious employment may result in poor social and material living conditions [8].

Multiple job holding (MJH) is another non-standard employment arrangement that has become more common in recent years. MJH is defined as having more than one paid job, either as an employee (combination MJH) or as an employee while also being self-employed (hybrid MJH). MJH is most common in Nordic countries (around 8% of the working population in Denmark, Norway, and Sweden, around 12% in Iceland) and the Netherlands (around 8% of the working population) [9].

Previous research on the relation between MJH and health has found mixed results. Some studies have found that MJH is related to worse health, e.g. higher mortality rates, higher risk of injuries, and higher risk of sickness absence due to mental health problems [10–12]. Other studies have found no relation between MJH and health-related outcomes such as long-term sickness absence and absence due to work-related accidents [13,14]. Furthermore, some studies have suggested that multiple job holders experience better mental health than single job holders [15,16]. An explanation may be that multiple job holders form a heterogeneous group of workers and that for some multiple job holders MJH is associated with better health, while for others MJH is associated with worse health. This is illustrated by a cross-sectional study in the Netherlands, which found that multiple job holders experienced less burn-out symptoms than single job holders, but that this only applied to those who did not have multiple jobs out of financial necessity [15]. In addition, another Dutch study distinguished four groups of multiple job holders, i.e. a vulnerable group, an indifferent group, a satisfied combination group and a satisfied hybrid group. Multiple job holders in the vulnerable group relatively often faced precarious employment conditions, e.g. temporary contracts and low job control. These vulnerable multiple job holders experienced worse physical and mental health than the other three groups [17].

The aim of this study is to increase our understanding of the relation between MJH and health. Therefore, it is important to study whether a negative relation between MJH and health is the result of factors associated with MJH, e.g. stress due to combining of work schedules [18], or factors associated with precarious employment, e.g. having a temporary contract. A possibility to investigate this, is studying the relation between MJH and health among workers in precarious employment. If, among workers in precarious employment, multiple job holders experience worse health than single job holders, this is an indication that MJH influences health independently from the degree of precariousness of individual jobs.

It is possible that precarious employment is more strenuous for multiple job holders than for single job holders. An important indicator of precarious employment is limited job control, for instance regarding working hours [1]. Among multiple job holders this may result in more stress than among single job holders, because limited control of working hours may make combining different work schedules more difficult [18,19].

It is important to study whether multiple job holders experience worse health than single job holders, since MJH is an increasingly common phenomenon in many countries. Moreover, if MJH increases the likeliness of poor health among employees in precarious employment, specific interventions and policies supporting these multiple job holders may be needed. Therefore, our main research question is: do differences in health exist between multiple and single job holders in precarious employment? In answering this research question, we will account for heterogeneity among multiple job holders. To do so, we will study whether differences in health exist between combination multiple job holders and hybrid multiple job holders on the one hand, and single job holders on the other hand? Distinguishing these groups is important since previous research has suggested that combination multiple job holders are overrepresented in groups of multiple job holders that experienced lower physical and mental health [17]. In addition, hybrid MJH may provide employees with better opportunities to combine jobs, e.g. with respect to combining work schedules [20]. Because previous research has shown that women are overrepresented in groups of workers who have multiple jobs for financial reasons [17], and that having a financial reason for MJH is associated with worse mental health [15] we tested interaction terms between MJH and gender and between MJH and household financial situation.

## Methods

### Study population

Participants were selected from the 2012 Netherlands Working Conditions Survey (NWCS) [21]. The NWCS is a yearly national survey on the labour situation of Dutch employees. In October 2012, a random sample of 80,000 persons aged 15 to 64 years were invited to participate in the NWCS. In this sample young persons and those with a migration background were overrepresented, because in previous years these groups of employees had lower response rates. Respondents could participate by filling out a hard copy of the questionnaire, or by filling out the online version of the questionnaire. To stimulate participation, respondents could join a lottery or donate money to the Red Cross. After two months and two reminders (the first after four weeks and the second after seven weeks), 25,223 employees participated (response 31.5%). To exclude students with side jobs from the present study, participants under the age of 25 years were excluded (N = 2,692). In addition, we excluded employees who were employed via the Sheltered Employment Act (SEA; through this act, persons with a handicap can work in a social work organization that adapts working conditions to their capabilities), to increase the homogeneity of the study sample (n = 177).

From the NWCS, employees in precarious employment were selected building on the operationalization of precariousness developed by Van Aerden et al. [1,3]. They have distinguished seven dimensions of precariousness: (1) employment stability; (2) material rewards; (3) workers’ rights and social protection; (4) working time arrangements; (5) employability opportunities; (6) collective organization; and (7) interpersonal power relations [1,3]. Table 1 presents the seven dimensions of precariousness identified by Van Aerden et al as well as the indicators used by them to measure these dimensions [1,3]. In addition, it presents the indicators used to measure precariousness in the present study. In total, 3609 employees in precarious employment were selected for this study. For more detailed information on how these respondents were selected, see the analyses section.

**Table 1:** Indicators used to identify group of employees in precarious employment.

Dimension of employment quality/precariousness	Indicators used by Van Aerden et al (2014, 2015, 2016)	Indicators in the present study	
		Variable in NWCS	Variable as included in latent class analysis
Employment stability	Type of employment contract • Permanent • Temporary > 1 year • Temporary < 1 year • Temporary agency	What is the nature of your employment contract 1. Employee with permanent contract 2. Employee with temporary contract with a prospect of a permanent contract 3. Employee with a temporary contract 4. Agency work 5. On call work 6. Working via the Act on Social Work Provision	Contract type: 1. Permanent 2. Temporary with prospect of permanent 3. Temporary 4. Agency/on call
Material rewards	Income level (country specific quartiles)	Satisfaction with salary • Very satisfied • Satisfied • Unsatisfied	Identical
	Non-wage benefits	-	-
Workers’ rights and social protection	Uncompensated exceptional working times	1. Do you work overtime, meaning more hours than it says in your contract? a. Yes, structurally b. Yes, incidentally c. No, never 2. Do you get paid for your overtime? a. Yes, for all over time b. Yes, for some overtime c. No	Both variables were combined to create one variable on overtime with the following categories: 1. No overtime 2. Paid overtime 3. Unpaid overtime
Working time arrangements	Schedule unpredictability	No separate variable, but the variable on contracts was used as a proxy (agency work and on call work are seen as unpredictable).	-
	Working hours	1. How many hours per week do you work according to your contract? (in the primary job) 2. How many hours per week would you want to work according to your contract?	Based on these two variables one variable with the following categories was constructed: 1. Full time (>34 hours) 2. Voluntary part time (<35 hours and does not want to work more hours) 3. Involuntary part time (<35 hours and wants to work more hours)
Employability opportunities	Training opportunities	Satisfaction with possibilities to learn: 1. Very satisfied 2. Satisfied 3. Unsatisfied	Identical
Collective organization	Information on OSH issues	*-*	-
	Working times setting procedure	Satisfaction with the possibility to determine working hours 1. Very satisfied 2. Satisfied 3. Unsatisfied	Identical
		Does your company have a collective labour agreement? 1. Yes 2. No 3. I don’t know	Identical
Interpersonal power relations	Employee involvement	Autonomy (continuous)	Divided into tertiles: • Low • Medium • High
	Unwanted behaviour	Can you indicate to which extent you have experienced in the last 12 months: 1. Unwanted sexual behavior from supervisors or colleagues? 2. Intimidation by supervisors or colleagues? 3. Physical violence by supervisors or colleagues? 4. Bullying by supervisors or colleagues	Dichotomized: • No • Yes

### Multiple job holding

MJH was measured using two questions. The first question asked respondents whether they had a paid job. Possible answers were: (1) no paid job; (2) one paid job; and (3) multiple paid jobs. The second question asked them if they had any income from other sources. Possible answers to this question were: (1) no; (2) yes, from my own business; (3) yes, from (early) retirement; (4) yes, from social benefits; and (5) yes, other. Respondents who answered that they had multiple jobs and/or had income from their own business next to having a paid job were categorized as multiple job holders. Two variables were created. One dichotomous variable (multiple job holders versus single job holders), and one categorical variable, consisting of three categories (combination MJH, hybrid MJH, and single job holders).

### Health

General self-perceived health was measured using one question “What do you think, in general, of your health?”. Answer categories were excellent, very good, good, reasonable, and bad. This variable was dichotomized (excellent, very good, and good versus reasonable and bad). Burnout complaints was measured using an adaption of the Utrecht Burnout Scale (UBOS) [22], which consists of five questions (e.g. “I feel emotionally exhausted by my work”). Answer categories for each of the questions were: (1) never, (2) a few times per year, (3) monthly, (4) a few times per month, (5) weekly, (6) a few times per week, and (7) every day. The overall score was computed by averaging the score on the five items. As a result, the overall score also ranged from 1 to 7. All respondents with a score lower than 3.2 were considered as not having a burnout complaints, those with a score of 3.2 or higher as having burnout complaints [22]. The internal consistency of the UBOS is good and the stability is reasonably good [23]. Construct validation of the adaption of the UBOS was conducted, and its construct validity was found to be good [24]. Respondents filled out a question on a wide range of chronic health problems. If respondents reported problems with hands or arms, problems with legs or feet, or problems with neck or back they were classified as having chronic musculoskeletal health problems. Respondents were also asked whether or not and how many days they had been absent from work due to sickness in the past 12 months. Because of the skewed distribution of the this variable, we chose to dichotomize it. Five days was taken as the cut-off value, because it correspondents to a (Dutch) work week.

### Confounders and covariates

We included demographic factors (gender, age, and educational level) as confounders. In addition, we included and variables used in the LCA if they differed significantly between single and multiple job holders (in general, CMJH or HMJH), because some indicators of precarious employment have been shown to be related to MJH and health [25–27]. The included variables were: contract type, involuntary part time work, uncompensated overtime, ability to determine working hours, autonomy, and bullying.

### Analyses

To identify employees in precarious employment Jung and Wickrama’s approach to latent class analysis (LCA) was used [24]. In short, we started with a single class model. Subsequently, a two class model was specified. The latter model was preferred to the former if: (1) the Bayesian information criterion was lower; (2) the bootstrap likelihood ratio test (BLRT) was statistically significant; (3) the average of posterior probabilities in each of the subgroups was higher than 0.8; (4) each of the subgroups contained more than 225 participants (1% of N = 22,354); and (5) the solution was interpretable and theoretically viable. If the two class model was preferred, a three class model was specified and compared to the two class model using the same criteria. This process was repeated until a newly specified model did not meet the criteria, in which case the last model to have met the criteria was chosen as the final model. In all models the indicators to measure precariousness specified in Table 1 were included. In the final model one class was selected as employees in precarious employment, based on their scores on the variables included in the LCA. The LCA was conducted in MPlus version 7.11.

To describe the study population we used descriptive statistics. Differences between multiple job holders and single job holders were tested for statistical significance using chi^2^ tests. The relation between MJH and health outcomes was analyzed cross-sectionally, using logistic regression analyses. First, univariable analysis were conducted. Second, multi-variable analyses were conducted, in which: (1) any demographic factors (age, gender and educational level) which differed between single and multiple job holders, i.e. gender, educational level and age; and (2) any variables included in the LCA that differed significantly between single and multiple job holders were included, i.e. contract type, involuntary part time work, uncompensated overtime, ability to determine working hours, autonomy, and bullying. Both steps were performed separately for the dichotomous MJH variable (MJH versus SJH) and for the categorical variable (CMJH and HMJH versus SJH). We tested interaction-terms between MJH and gender and between MJH and household financial situation. All analyses were conducted using SPSS version 25.

### Ethics

Potential respondents of NWCS were informed about the study in a letter accompanying the questionnaire. All data was pseudonymized before access was provided, i.e. all directly identifying personal details were replaced by a pseudo key. The Medical Ethics Review Committee of VU University Medical Center declared that the present study is not subjected to the Dutch Medical Research Involving Human Subjects Act.

## Results

Table 2 shows the results of the LCA performed to select employees in precarious employment. Model 4 was preferred to the other models. Model 2 had a BIC lower than model 1, but no clear group of employees in precarious employment could be distinguished in this model. Model 3, 4, and 5 all had a lower BIC than the previous model. However, these models also resulted in at least two groups with a posterior probability lower than 0.8. We preferred model 4 to model 3, because the BIC was lower. We preferred model 4 to model 5, because the relative number of groups with a posterior probability lower than 0.8 was lower in model 4. In addition, in model 5 no clear group of employees in precarious could be distinguished, in contrast to model 4. An overview of the four groups identified in model 4 is presented in the S1 Table.

**Table 2:** Results of the latent class analysis.

	Bayesian Information Criterion	Number of respondents in each category	Posterior probabilities	Bootstrap LRT
Model 1	329998.420	1. 22354	1. 1.000	-
Model 2	320886.947	1. 12999 2. 9355	1. 0.895 2. 0.847	0.000
Model 3	318816.706	1. 6442 2. 10081 3. 5831	1. 0.798 2. 0.757 3. 0.853	0.000
Model 4*	317214.725	1. 3609 2. 7887 3. 3313 4. 7545	1. 0.774 2. 0.738 3. 0.848 4. 0.833	0.000
Model 5	316106.954	1. 3206 2. 7325 3. 7424 4. 3279 5. 1120	1. 0.757 2. 0.715 3. 0.822 4. 0.848 5. 0.776	0.000


* Model 4 was selected for this study

**Fig 1:**
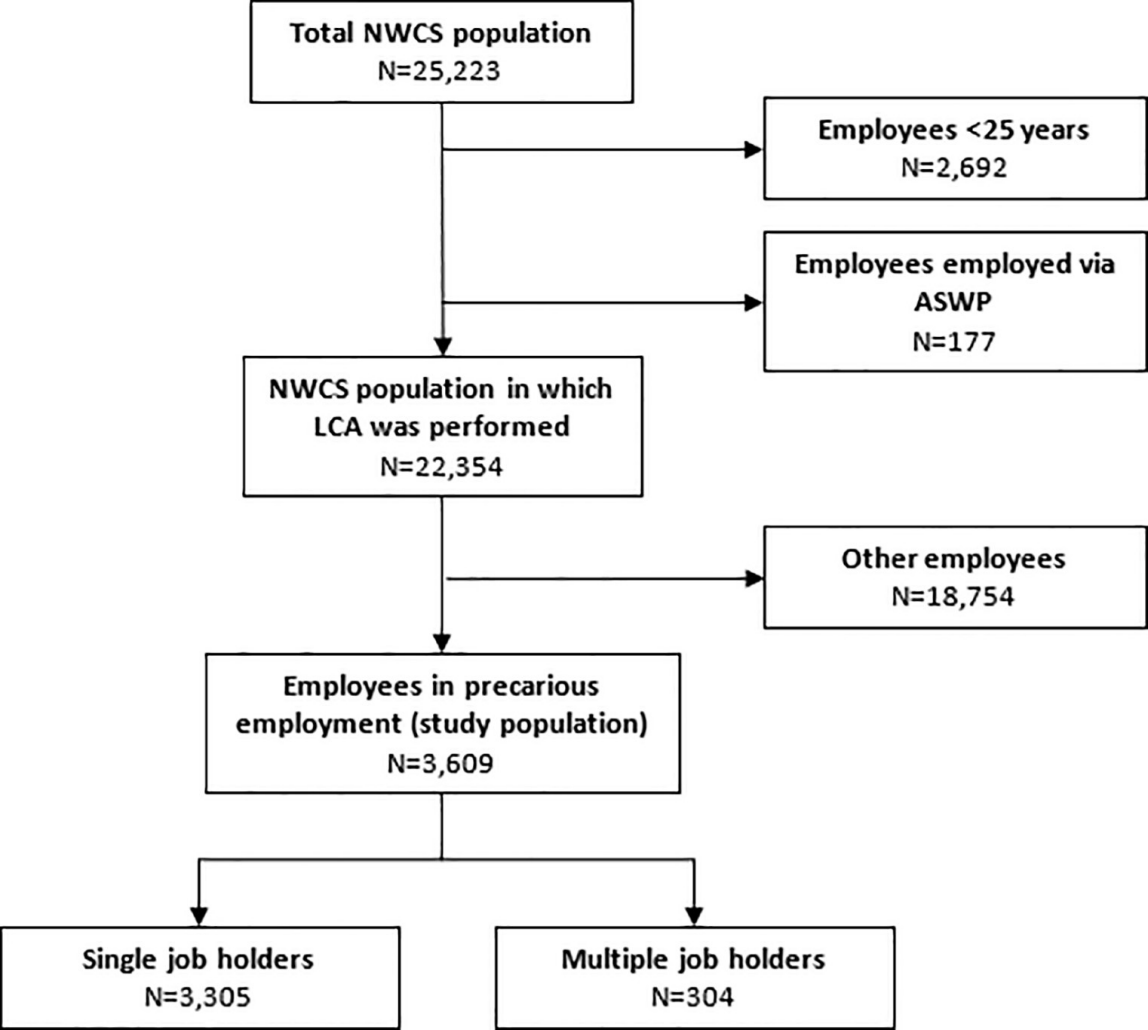
Study population.

Of the four groups, one showed characteristics of employees in precarious employment, and was selected for this study. The group consisted of 3,609 employees, among whom 304 multiple job holders (8%) (see Fig 1 for an overview of the selection of the study population). Among employees in precarious employment, multiple job holders were more often female, aged 45–54 years, and higher educated compared to single job holders (see Table 3). The mean age of all participants was 43 years. No age differences were found between multiple and single job holders. Furthermore, multiple job holders less often had a permanent contract, more often worked part-time involuntarily, and were more often satisfied with the opportunity to determine their working hours.

**Table 3:** Description of the study population.

	Employees in precarious employment									
	All		Single job holders		Multiple job holders					
					All		Combination		Hybrid	
	n = 3609		n = 3305		n = 304		n = 174		n = 130	
	n	%	n	%	n	%	n	%	n	%
**Demographic factors**										
Female	1803	50%	1627	49%	176	58%^a^	115	66%^b^	61	47%^c^
Age										
*25–34*	951	26%	874	27%	77	24%^a^	47	27%	30	23%^c^
*35–44*	875	24%	805	24%	70	23%	38	22%	32	25%
*45–54*	1107	31%	993	30%	114	38%	60	35%	54	42%
*55–64*	676	19%	633	19%	43	14%	29	17%	14	11%
Educational level										
*Low*	857	24%	813	25%	44	15%^a^	32	18%^b^	12	9%^c^
*Medium*	1598	45%	1471	45%	127	42%	71	41%	56	43%
*High*	1134	32%	1001	31%	133	44%	71	41%	62	48%
**Variables in latent class analysis**										
Contract										
*Agency/oncall*	274	8%	231	7%	43	14%^a^	29	17%^b^	14	11%
*Temporary*	372	10%	331	10%	41	14%	26	15%	15	12%
*prospect of permanent*	213	6%	194	6%	19	6%	11	6%	8	6%
*Permanent*	2733	76%	2533	77%	200	66%	107	62%	93	72%
Salary										
*Unsatisfied*	2184	61%	2016	62%	168	56%	93	54%	75	59%
*Satisfied*	1282	36%	1159	35%	123	41%	74	43%	49	38%
*Very satisfied*	112	3%	102	3%	10	3%	6	4%	4	3%
Uncompensated overtime										
*No overtime*	981	28%	908	28%	73	24%	39	23%^b^	34	26%
*Compensated overtime*	1412	40%	1275	39%	137	46%	85	50%	52	40%
*Uncompensated overtime*	1180	33%	1090	33%	90	30%	46	27%	44	34%
Working hours										
*Full-time*	1984	55%	1881	57%	103	34%^a^	49	29%^b^	54	43%^c^
*Voluntary part-time*	777	22%	704	21%	73	24%	37	22%	36	28%
*Involuntary part-time*	829	23%	706	22%	123	41%	86	50%	37	29%
Training opportunities										
*Unsatisfied*	2201	62%	2005	61%	196	65%	109	63%	87	68%
*Satisfied*	1227	34%	1132	35%	95	32%	57	33%	38	30%
*Very satisfied*	140	4%	130	4%	10	3%	7	4%	3	2%
Collective labour agreement										
*Don’t know*	425	12%	380	12%	45	15%	26	15%	19	15%
*No*	527	15%	482	15%	45	15%	22	13%	23	18%
*Yes*	2623	73%	2410	74%	213	70%	126	72%	87	67%
Ability to determine working hours										
*Unsatisfied*	2152	61%	1994	62%	158	53%^a^	91	53%	67	52%
*Satisfied*	1238	35%	1111	34%	127	42%	74	43%	53	41%
*Very satisfied*	150	4%	135	4%	15	5%	7	4%	8	6%
Autonomy										
*Low*	1942	54%	1779	54%	163	54%	103	60%^b^	60	47%
*Medium*	1390	39%	1282	39%	108	36%	52	30%	58	44%
*High*	256	7%	226	7%	30	10%	18	10%	12	9%
Bullying (yes)	1534	43%	1416	43%	118	39%	55	32%^b^	63	49%
**Health**										
General self-perceived physical health (bad/reasonable)	709	20%	656	20	53	18%	32	19%	21	16%
Burnout complaints	1072	30%	994	30%	78	26%	40	23%	38	29%
Musculoskeletal health	810	23%	741	23%	69	23%	42	25%	27	21%
Sickness absence ≥ 5 days	1212	34%	1131	35%	81	27%	41	24%	40	31%

^a^ Statistically significant difference between multiple job holders and single job holders in precarious employment and other employees.

^b^ Statistically significant difference between combination multiple job holders single job holders in precarious employment

^c^ Statistically significant difference between hybrid multiple job holders single job holders in precarious employment

The results of the analyses of the differences in health between multiple job holders and single job holders showed no statistically significant differences regarding general self-perceived health, burnout complaints, and chronic musculoskeletal health problems (see Table 4). In crude analyses multiple job holders less often experienced sickness absence than single job holders (see Table 4). In fully adjusted analyses, this difference was no longer statistically significant.

**Table 4:** Health differences between multiple and single job holders in precarious employment (single job holding is reference category).

	MJH (total)			MJH (combination)			MJH (hybrid)		
	OR	95% CI	p-value	OR	95% CI	p-value	OR	95% CI	p-value
Poor self-perceived general health									
Model 1	0.85	0.63–1.16	0.312	0.91	0.62–1.35	0.650	0.78	0.48–1.25	0.293
Model 2	0.87	0.64–1.19	0.381	0.93	0.62–1.38	0.702	0.80	0.49–1.29	0.353
Model 3	0.91	0.65–1.26	0.561	1.06	0.69–1.61	0.803	0.74	0.44–1.23	0.242
Burn-out complaints									
Model 1	0.80	0.61–1.05	0,103	0.69	0.48–0.99	**0.046**	0.96	0.65–1.41	0.826
Model 2	0.77	0.59–1.02	0.064	0.68	0.47–0.98	**0.038**	0.91	0.62–1.34	0.626
Model 3	0.88	0.65–1.18	0.383	0.83	0.56–1.23	0.355	0.94	0.61–1.43	0.760
Chronic musculoskeletal health problems									
Model 1	1.02	0.77–1.36	0.876	1.12	0.78–1.61	0.531	0.90	0.58–1.39	0.631
Model 2	1.07	0.80–1.43	0.638	1.16	0.80–1.67	0.436	0.96	0.62–1.50	0.865
Model 3	1.09	0.81–1.48	0.570	1.22	0.83–1.80	0.317	0.94	0.59–1.49	0.794
Sickness absence ≥5 days									
Model 1	0.69	0.53–0.90	**0.006**	0.58	0.41–0.84	**0.003**	0.85	0.58–1.24	0.386
Model 2	0.71	0.54–0.92	**0.011**	0.59	0.41–0.85	**0.004**	0.88	0.60–1.29	0.519
Model 3	0.78	0.59–1.04	0.087	0.70	0.47–1.03	0.067	0.89	0.59–1.34	0.571

Model 1: Crude

Model 2: Adjusted for gender, age, and educational level

Model 3: Adjusted for model gender, age, educational level, contract, involuntary part time work, uncompensated overtime, ability to determine working hours, autonomy, and bullying

Bold p-values are <0.005

We found no significant differences between hybrid multiple job holders and single job holders regarding any of the outcome measures (see Table 4). We did find that combination multiple job holders experienced burnout complaints and sickness absence less often than single job holders in crude analyses (see Table 4). In fully adjusted analyses, these differences were no longer statistically significant.

The interaction-terms between MJH and gender and between MJH and household financial position were not statistically significant in any of the analyses.

## Discussion

The main aim of this study was to investigate whether health differences exist between multiple and single job holders in precarious employment. We found no differences in self-perceived general health, burnout complaints, chronic musculoskeletal health problems, and sickness absence in fully adjusted analyses. In addition, we aimed to investigate differences in health between combination multiple job holders, hybrid multiple job holders and single job holders. We found no differences between hybrid multiple job holders and single job holders. We did find that combination multiple job holders experienced sickness absence less often than single job holders, but this difference was no longer statistically significant in adjusted analyses.

The finding that MJH in general was not associated with any of the health outcomes is in line with a previous study among Danish employees, which found that MJH in general is not associated with long-term sickness absence [13]. However, other previous studies, in the US as well as in the Netherlands, have found an association between MJH in general and various health outcomes [16,28]. An explanation for this contrasting finding may be that, in the present study, the focus was on employees in precarious employment.

In addition, the finding that combination multiple job holders experienced sickness absence less often than single job holders was borderline significant. This is in contrast to a previous Danish study, which found that, among employees who worked full-time or more, combination multiple job holders experienced long-term sickness absence more often [13]. These contrasting findings may be explained by a difference in the outcome measure, i.e. respectively five days or more versus five (consecutive) weeks or more. It has been suggested that the relationship between health and long-term sickness absence is stronger than between health and short-term sickness absence [29]. In addition, these contrasting findings may be explained by differences in study population: in the present study, employees working part-time were overrepresented, while in the Danish study, a relation between combination MJH and sickness absence was only found among those working full-time or more.

Based on the results of this study, we found no indications that multiple job holders in precarious employment experience worse health than single job holders in precarious employment, despite potential health risks associated with MJH. A possible explanation may be that the influence of MJH on health in addition to the influence of precarious employment on health is relatively small. Most studies on precarious employment suggest that it is associated with poorer health (3,5,6), whereas previous research on MJH and health showed mixed results. Further, previous research has suggested that MJH may also positively influence health, e.g. adding an extra job can be used as a strategy to reduce income insecurity [18]. In the present study, we found that multiple job holders more often worked part-time involuntarily and more often had a temporary contract than single job holders. Among employees in precarious employment, MJH may thus be used to reduce income insecurity, which may positively influence health [30]. It is recommended that future research studies the relation between MJH and health in other groups of employees, since the relation between MJH and health may be different among employees who are not in precarious employment.

The main strength of this study is that it is based on a large, representative sample of Dutch employees. Consequently, the results can be considered representative for Dutch employees in precarious employment. Another strength is that in the present study we were able to incorporate the multi-dimensional nature of precariousness by using LCA to select employees in precarious employment. Previous research has often focused on only one dimension to measure precariousness employment [31]. This study suffers from a number of limitations. Firstly, in previous research different definitions and operationalizations of precarious employment have been used [8], for instance including physical job demands [32]. It is possible that a different operationalization of precarious employment would have resulted in a different study population, and therefore in different results. In addition, because LCA was used to identify a group of employees in precarious employment, some employees with a permanent employment were classified as being in precarious employment, which may be counterintuitive. However, in many studies, including the present study, precarious employment is seen as a multi-dimensional construct [5]. Therefore, employees who have a permanent contract, but face low material rewards, low control regarding working hours, and low social protection, for instance, can still be considered as being in precarious employment. Secondly, the indicators of precariousness used in the present study deviate slightly from the indicators used by Van Aerden. For instance, for some dimensions no (adequate) indicators were available (non-wage benefits, schedule unpredictability). In addition, unfortunately, in the present study objective indicators were not available for all dimensions of precariousness, e.g. salary and opportunities to influence working times. Subjective indicators, for instance for salary, may also measure other job and personal characteristics, such as appreciation of the job in relation to salary, and whether the salary is enough to cover the expenses of the household. Therefore, these measures are less suitable to measure the precariousness of a job. Because subjective measures of income have been shown to be related more strongly to health related outcomes, such as quality of life [33], this may have affected our findings. Further, we only had information on working hours in the first job. Therefore, we were not able to include total working hours in the analyses, e.g. as a potential confounder or effect-modifier. The number of working hours may influence the demands of MJH, and may be an indicator of MJH-groups. Previous research suggests that satisfied multiple job holders that experience better health work more hours on average [17]. Therefore influence the relation between MJH and health. Thirdly, the cross-sectional design of the present study does not allow for causal inferences to be made. Future longitudinal research is recommended to investigate whether health selection in and out of multiple job holding plays a role among workers in precarious work. Fourthly, we could only make a distinction between combination and hybrid MJH to account for heterogeneity among multiple job holders. Distinguishing between groups of multiple job holders based on their reason for MJH, experience with MJH, and working hours may have given more insight into heterogeneity among multiple job holders, although we believe this heterogeneity to be limited in the present study, because of the relatively homogenous study population. Fifthly, despite the large study sample, the relatively small number of respondents who reported having multiple jobs limited the statistical power of this study. As the effect sizes found in this study were rather small, a larger sample may have resulted in statistically significant differences, but it is questionable whether these differences should be considered as relevant. Further, the present study was conducted among employees in the Netherlands. It is possible that a similar study in a different country would find different results. It has been suggested, for instance, that more egalitarian labour institutions have better population health outcomes [4]. Additionally, social security systems may influence the reason why employees have multiple jobs and how they experience it. In more generous social security systems, having multiple jobs out of financial necessity may be less common, for instance. This may in turn influence the relation between MJH and health among employees in precarious employment.

In conclusion, we did not find health differences between multiple job holders in general, hybrid multiple job holders and single job holders among employees in precarious employment. Longitudinal research is necessary to determine whether specific? policies aimed at multiple job holders in precarious employment are needed. In addition, further research on the relation between MJH and health in (other) groups of employees and in other countries is recommended to increase our knowledge on the relation between MJH and health.

## Supporting information

S1 Table Four groups of employees identified in the latent class analysis. (DOCX)
