# Supplementary material for: Health differences between multiple and single job holders in precarious employment in the Netherlands: A cross-sectional study among Dutch workers
Source: PLoS One. 2019 Sep 11;14(9):e0222217. doi: 10.1371/journal.pone.0222217 (PMC6738659; doi:10.1371/journal.pone.0222217)
Supplement: S1 Table — (DOCX) [file pone.0222217.s001.docx]

**S1 Table. Four groups of employees identified in the Latent Class Analysis**

|  | **Precarious employment** | **High security, low autonomy** | **Standard Employment** | **High security, high autonomy** |
| --- | --- | --- | --- | --- |
|  | **n = 3609** | **n = 7887** | **n = 3313** | **n = 7545** |
|  |  |  |  |  |
| Contract |  |  |  |  |
| Agency/oncall | 7% | 3% | 1% | 1% |
| Temporary | 9% | 4% | 4% | 4% |
| Prospect of permanent | 6% | 6% | 6% | 4% |
| Permanent | 78% | 88% | 89% | 91% |
| Salary |  |  |  |  |
| Unsatisfied | 54% | 17% | 5% | 16% |
| Satisfied | 42% | 74% | 36% | 79% |
| Very satisfied | 4% | 9% | 58% | 6% |
| Uncompensated overtime |  |  |  |  |
| Uncompensated overtime | 33% | 30% | 53% | 47% |
| Compensated overtime | 40% | 40% | 26% | 29% |
| No overtime | 27% | 30% | 21% | 25% |
| Working hours |  |  |  |  |
| Involuntary part-time | 21% | 12% | 6% | 8% |
| Voluntary part-time | 27% | 48% | 31% | 30% |
| Full-time | 52% | 40% | 63% | 62% |
| Trainig opportunities |  |  |  |  |
| Unsatisfied | 49% | 5% | 3% | 12% |
| Satisfied | 45% | 76% | 23% | 75% |
| Very satisfied | 7% | 19% | 74% | 13% |
| Collective labour agreement |  |  |  |  |
| Don’t know | 11% | 5% | 5% | 4% |
| No | 13% | 4% | 18% | 18% |
| Yes | 76% | 91% | 78% | 78% |
| Ability to determine working hours |  |  |  |  |
| Unsatisfied | 53% | 22% | 3% | 5% |
| Satisfied | 42% | 76% | 23% | 72% |
| Very satisfied | 5% | 2% | 75% | 24% |
| Autonomy |  |  |  |  |
| Low | 53% | 51% | 8% | 0% |
| Medium | 37% | 49% | 16% | 19% |
| High | 11% | 0% | 76% | 81% |
| Bullying (yes) | 37% | 12% | 7% | 12% |
